# ImageGP 2 for enhanced data visualization and reproducible analysis in biomedical research

**DOI:** 10.1002/imt2.239

**Published:** 2024-09-12

**Authors:** Tong Chen, Yong‐Xin Liu, Tao Chen, Mei Yang, Siqing Fan, Minglei Shi, Buqing Wei, Huijiao Lv, Wandi Cao, Chongming Wang, Jianzhou Cui, Jiwen Zhao, Yilai Han, Jiao Xi, Ziqiang Zheng, Luqi Huang

**Affiliations:** ^1^ State Key Laboratory for Quality Ensurance and Sustainable Use of Dao‐di Herbs, National Resource Center for Chinese Materia Medica China Academy of Chinese Medical Sciences Beijing China; ^2^ Genome Analysis Laboratory of the Ministry of Agriculture and Rural Affairs, Agricultural Genomics Institute at Shenzhen Chinese Academy of Agricultural Sciences Shenzhen China; ^3^ Northwest Institute of Plateau Biology Chinese Academy of Sciences Xining Qinghai China; ^4^ Lushan Botanical Garden Chinese Academy of Sciences Jiujiang China; ^5^ School of Life Course and Population Sciences King's College London London UK; ^6^ State Key Laboratory of Efficient Utilization of Arid and Semi‐arid Arable Land in Northern China, The Institute of Agricultural Resources and Regional Planning Chinese Academy of Agricultural Sciences Beijing China; ^7^ Key Laboratory of Human Disease Comparative Medicine, National Health Commission of China (NHC), Institute of Laboratory Animal Science, Chinese Academy of Medical Sciences Peking Union Medicine College Beijing China; ^8^ Nanjing Agricultural University Nanjing China; ^9^ College of Resources and Environment Huazhong Agricultural University Wuhan Hubei China; ^10^ Immunology Translational Research Program, Yong Loo Lin School of Medicine National University of Singapore Singapore Singapore; ^11^ Immunology Program, Life Sciences Institute National University of Singapore Singapore Singapore; ^12^ NUS‐Cambridge Immunophenotyping Centre National University of Singapore Singapore Singapore; ^13^ State Key Laboratory of Crop Stress Resistance and High‐Efficiency Production, College of Agronomy Northwest A&F University Yangling Shaanxi People's Republic of China; ^14^ Department of Neurology, National Center for Neurological Disorders Xuanwu Hospital Capital Medical University Beijing China; ^15^ College of Natural Resources and Environment Northwest A&F University Yangling Shaanxi China; ^16^ School of Life Science and Technology Wuhan Polytechnic University Wuhan China

**Keywords:** biology cloud platform, data analysis, data transformation, data visualization, ImageGP

## Abstract

ImageGP is an extensively utilized, open‐access platform for online data visualization and analysis. Over the past 7 years, it has catered to more than 700,000 usages globally, garnering substantial user feedback. The updated version, ImageGP 2 (available at https://www.bic.ac.cn/BIC), introduces a redesigned interface leveraging cutting‐edge web technologies to enhance functionality and user interaction. Key enhancements include the following: (i) Addition of modules for data format transformation, facilitating operations such as matrix merging, subsetting, and transformation between long and wide formats. (ii) Streamlined workflows with features like preparameter selection data validation and grouping of parameters with similar attributes. (iii) Expanded repertoire of visualization functions and analysis tools, including Weighted Gene Co‐Expression Network Analysis, differential gene expression analysis, and FASTA sequence processing. (iv) Personalized user space for uploading large data sets, tracking analysis history, and sharing reproducible analysis data, scripts, and results. (v) Enhanced user support through a simplified error debugging feature accessible with a single click. (vi) Introduction of an R package, ImageGP, enabling local data visualization and analysis. These updates position ImageGP 2 as a versatile tool serving both wet‐lab and dry‐lab researchers with expanded capabilities.

## Highlights

- Advanced user interface, expanded analytical capabilities, and seamless data handling.
- New modules for data transformation and preparameter selection data validation.
- Personalized user center, reproducible scripts, seamless error debugging, the introduction of local analysis capabilities.

## Graphical abstract

The infinity symbol illustrates the seamless workflow of ImageGP 2, encompassing essential functions such as data format transformation, data validation, and parameter combination. This process culminates in the generation of diverse visual outputs, including line, point, and bar plots. Key features include a personalized user center for managing large data sets, interactive visualizations, and streamlined error feedback mechanisms. Additionally, the introduction of the ImageGP R package enables local and batch analyses. Overall, the infinity symbol embodies the limitless potential for data analysis and visualization offered by ImageGP 2.

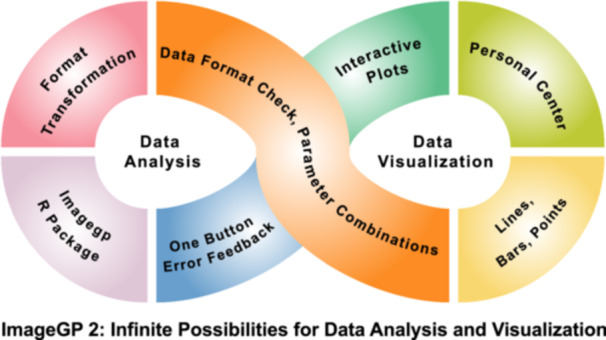

## INTRODUCTION

In the era of “Omics” in life sciences, the accumulation of vast and complex biological data sets has become ubiquitous across genomic, transcriptomic, epigenomic, proteomic, metabolomic, and clinical domains [1]. The big science projects such as the Human Genome Project [2, 3], ENCODE [4], Human Cell Atlas [5], Earth BioGenome Project [6], Protist 10,000 Genomes Project [7], and the Proteomic Navigator of the Human Body (π‐HuB) are generating unprecedented volumes of data. For instance, GenBank's latest release (261.0) alone encompasses over 3.38 billion whole genome sequencing records, containing 27.9 trillion base pairs of genome data [8]. Similarly, the NGDC GSA warehouse holds nearly 50 petabytes of data (June 2024) [9], while IMP catalogs 716 billion genome base pairs from plant genomes [10]. The management and integration of such heterogeneous data pose significant challenges, necessitating robust tools and methodologies.

Effective utilization of these vast biological data sets promises substantial value but requires overcoming numerous challenges, including data complexity, integration across diverse resources, and the establishment of standardized principles for big data handling [11, 12, 13, 14]. Tools facilitating the analysis of large biological data sets play a pivotal role in translating this wealth of information into comprehensive insights into biomedical mechanisms, which are crucial for applications in translational and personalized medicine [1].

In this context, data visualization emerges as a crucial tool for researchers striving to comprehend and communicate complex biological insights effectively. Data visualization tools, particularly those in the form of interactive dashboards, offer intuitive graphical representations such as charts, graphs, and maps. These visual aids enable researchers to discern trends, identify outliers, and uncover patterns within data sets, thereby enhancing understanding and facilitating data‐driven decision‐making. The accessibility and interpretability offered by visual representations transcend the barriers posed by raw data, making complex scientific findings accessible to broader audiences.

Numerous tools have been developed to cater to the diverse needs of data visualization. These tools can broadly be categorized into command‐line tools (e.g., R, Python, Perl, LaTeX, Javascript, MATLAB, Gnuplot, Graphviz), desktop software (e.g., Excel, PowerPoint, Cytoscape [15], Gephi [16], IGV [17], Mayavi [18], Tbtools [19]), and online platforms (e.g., ImageGP [20], EVenn [21], HemI [22], Sangerbox [23], OmicStudio [24], shinyCircos [25], TOmicsVis [26], Wekemo Bioincloud [27]), iMeataLab Suite [28], iNAP [29], Majorbio Cloud [30], MetOrigin [31]. Each category offers distinct advantages and limitations, balancing flexibility, ease of use, and computational resources.

ImageGP 2 (accessible at https://www.bic.ac.cn/BIC/#/) represents a significant evolution in online data visualization and analysis platforms, tailored to meet the advanced needs of biomedical researchers. This updated version introduces a redesigned interface utilizing state‐of‐the‐art web technologies, enhancing functionality and user interaction. Key features include modules for data format transformation, streamlined workflows with preparameter selection validation, an expanded array of visualization functions and analytical tools (e.g., Weighted Gene Co‐Expression Network Analysis [WGCNA], differential gene expression analysis), and personalized user spaces for managing large data sets and analysis history. Additionally, the integration of an R package, ImageGP, extends these capabilities to local environments, addressing challenges related to usability and data management in both wet‐lab and dry‐lab settings.

## RESULT

### Overview of ImageGP 2

**Figure 1:**
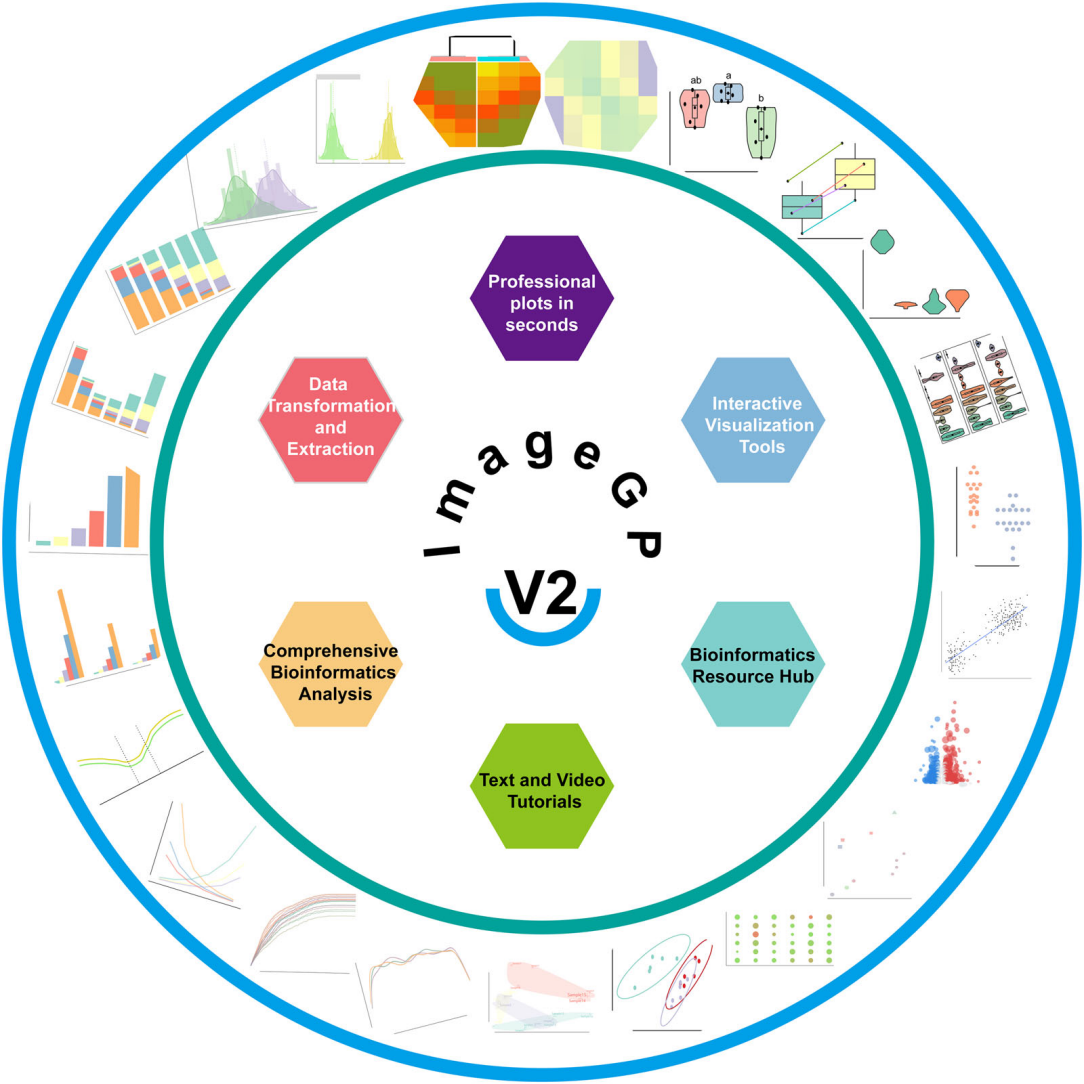
Functional framework of ImageGP 2. The inner circle illustrates six key sections: professional plot generation, data transformation and extraction, bioinformatics analyses, interactive visualization tools, a bioinformatics resource hub, and text and video tutorials. The outer circle presents examples of representative visualization outputs.

ImageGP 2 represents a substantial redesign aimed at enhancing user experience and functionality based on feedback from its predecessor. It incorporates 45 distinct tools and includes 13 instructional resources in six thematic sections (Figure 1). These encompass professional plot generation, data transformation and extraction capabilities, bioinformatics analyses, interactive visualization tools, as well as text and video tutorials. A dedicated bioinformatics resource section is also provided.

The platform boasts 17 data visualization tools tailored for creating diverse plots such as heatmaps, box plots, bar plots, scatter plots (including variants like enrichment and volcano plots), Principal Co‐ordinates Analysis plots, histograms, line plots, and various Venn diagrams (Figure 1). While many of these tools were available in the earlier version of ImageGP, the backend codes have been entirely restructured. This overhaul introduces enhanced data validation logic to minimize user input errors and offers expanded parameter options to refine data screening and aesthetic attributes exploration. Comprehensive tutorials are available in both text and video formats, accessible within the tutorial section and alongside each tool for user convenience. Each tool features a carousel chart outlining input data formats, parameters, and output formats, accompanied by demo buttons to facilitate the reproducibility of illustrative examples.

### Addition of modules for data format transformation

In the context of biological analysis, the majority of processed data is stored in matrix formats, such as gene expression matrices and matrices detailing abundance levels of bacteria, proteins, or metabolites. Typically, these matrices adopt a wide format structure, suitable for comparisons across all samples; for example, shown in heatmap. However, visualization tools like ggplot2, grounded in The Grammar of Graphics, require data in a long format to effectively map variables to visual aesthetics [32].

**Figure 2:**
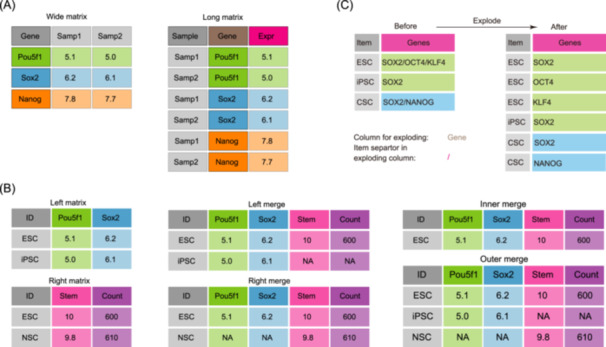
Matrix formats and transformations. (A) Illustration of the transformation between wide and long format matrices. (B) Overview of matrix merging capabilities, demonstrating five modes: left, right, inner, outer, and horizontal stacking (hstack). (C) Description of the “explode matrix” function, which expands matrix dimensions by splitting elements in a single column and duplicating values from other columns.

Semantically, a wide matrix should contain more columns and a long matrix must contain more rows (Figure 2A). This is not the main difference. A wide format matrix typically contains numerous columns where each column (except the first) holds homogeneous data. For instance, in a gene expression matrix, each numeric value within columns represents gene expression levels across samples. Utilizing this format for visualizations, such as mapping expression values to point size attributes, presents challenges due to the need to aggregate data across all columns.

Conversely, a long format matrix from the gene expression table is structured with fewer columns (e.g., samples, genes, values) and more rows, representing each unique combination of genes and samples (Figure 2A). This format accommodates heterogeneous data among columns, facilitating straightforward mapping of specific columns to distinct aesthetic attributes. Notably, a matrix may be treated as wide or long formats depending on the analytical requirements. For example, a gene expression matrix functions as a wide format when comparing all samples, yet serves as a long format for generating correlation plots between any two samples using scatter plots, where columns denote varied attributes.

Another common operation involves matrix merging, executed in five modes: left (retaining all items from the left matrix), right (retaining all items from the right matrix), inner (retaining common items from both matrices), outer (combining all items from both matrices), and hstack (concatenating all columns) (Figure 2B). This functionality is typically employed to integrate a long‐format abundance matrix with metadata matrices, thereby incorporating additional sample attributes. Furthermore, this operation supports matrix subset extraction using left, right, or inner modes. For instance, in the left merging mode, only the subset of expression data corresponding to target genes in the left matrix is extracted. Additionally, matrix merging facilitates tasks such as ID mapping for gene identification transfer.

Here, we utilize a gene expression plot to illustrate the application of these matrix operation functions. Typically, the gene expression matrix is structured in a wide matrix format, as shown below:

**Table:** 

Gene	Samp1	Samp2	Samp3	Samp4	Samp5	Samp6	Samp7	Samp8	Samp9	Samp10	Samp11	Samp12
Gene1	5.0	5.0	5.0	5.8	5.0	5.8	9.9	9.6	9.9	9.6	8.9	10.4
Gene2	9.9	8.0	4.2	4.7	4.2	4.7	9.9	8.6	9.9	8.6	4.2	4.2
Gene3	8.2	8.1	9.7	7.8	9.7	7.8	12.5	10.8	12.5	10.8	12.3	11.1
Gene4	6.5	7.1	6.8	6.9	6.8	6.9	11.1	10.8	11.1	10.8	10.5	11.1

Suppose we want to analyze the expression distribution of Gene1 across all samples using a density plot. Initially, this matrix is structured in a wide format. To facilitate analysis, we transpose the matrix:

**Table:** 

Samp	Gene1	Gene2	Gene3	Gene4
Samp1	5.0	9.9	8.2	6.5
Samp2	5.0	8.0	8.1	7.1
Samp3	5.0	4.2	9.7	6.8
Samp4	5.8	4.7	7.8	6.9
Samp5	5.0	4.2	9.7	6.8
Samp6	5.8	4.7	7.8	6.9
Samp7	9.9	9.9	12.5	11.1
Samp8	9.6	8.6	10.8	10.8
Samp9	9.9	9.9	12.5	11.1
Samp10	9.6	8.6	10.8	10.8
Samp11	8.9	4.2	12.3	10.5
Samp12	10.4	4.2	11.1	11.1

In this transposed matrix, one column represents a gene, transforming it into a long matrix suitable for gene‐centric analysis with each gene as one separate attribute. By pasting this data into the histogram plot tool and configuring parameters, we could generate the expression distribution profile for Gene1 in all samples (Figure S1).

To extend the analysis to compare gene expression profiles among different sample groups, we incorporate metadata:

**Table:** 

Samp	Group
Samp1	Root
Samp2	Root
Samp3	Root
Samp4	Root
Samp5	Root
Samp6	Root
Samp7	Leaf
Samp8	Leaf
Samp9	Leaf
Samp10	Leaf
Samp11	Leaf
Samp12	Leaf

Using the “merge matrix” tool, we combine these matrices to create a merged data set (Figure S2):

**Table:** 

Samp	Gene1	Gene2	Gene3	Gene4	Group
Samp1	5.0	9.9	8.2	6.5	Root
Samp2	5.0	8.0	8.1	7.1	Root
Samp3	5.0	4.2	9.7	6.8	Root
Samp4	5.8	4.7	7.8	6.9	Root
Samp5	5.0	4.2	9.7	6.8	Root
Samp6	5.8	4.7	7.8	6.9	Root
Samp7	9.9	9.9	12.5	11.1	Leaf
Samp8	9.6	8.6	10.8	10.8	Leaf
Samp9	9.9	9.9	12.5	11.1	Leaf
Samp10	9.6	8.6	10.8	10.8	Leaf
Samp11	8.9	4.2	12.3	10.5	Leaf
Samp12	10.4	4.2	11.1	11.1	Leaf

This merged data set allows us to conduct a comparative analysis between sample groups, visualizing the expression distribution profile of each gene across different conditions using still the histogram plot tool (Figure S3).

If we want to analyze multiple genes or all genes simultaneously, the matrix is not suitable since each gene is one individual attribute. One way to do this is to collapse all genes into one column and all expression values into one column. That is the function of the tool “Wide to long matrix” (Figure S4):

**Table:** 

Samp	Group	Gene	value
Samp1	Root	Gene1	5.0
Samp2	Root	Gene1	5.0
Samp3	Root	Gene1	5.0
Samp4	Root	Gene1	5.8
Samp5	Root	Gene1	5.0
Samp6	Root	Gene1	5.8
Samp7	Leaf	Gene1	9.9
Samp8	Leaf	Gene1	9.6
Samp9	Leaf	Gene1	9.9
Samp10	Leaf	Gene1	9.6
Samp11	Leaf	Gene1	8.9
Samp12	Leaf	Gene1	10.4
Samp1	Root	Gene2	9.9
Samp2	Root	Gene2	8.0
Samp3	Root	Gene2	4.2
Samp4	Root	Gene2	4.7
Samp5	Root	Gene2	4.2
Samp6	Root	Gene2	4.7
Samp7	Leaf	Gene2	9.9
Samp8	Leaf	Gene2	8.6
Samp9	Leaf	Gene2	9.9
Samp10	Leaf	Gene2	8.6
Samp11	Leaf	Gene2	4.2
Samp12	Leaf	Gene2	4.2
Samp1	Root	Gene3	8.2
Samp2	Root	Gene3	8.1
Samp3	Root	Gene3	9.7
Samp4	Root	Gene3	7.8
Samp5	Root	Gene3	9.7
Samp6	Root	Gene3	7.8
Samp7	Leaf	Gene3	12.5
Samp8	Leaf	Gene3	10.8
Samp9	Leaf	Gene3	12.5
Samp10	Leaf	Gene3	10.8
Samp11	Leaf	Gene3	12.3
Samp12	Leaf	Gene3	11.1
Samp1	Root	Gene4	6.5
Samp2	Root	Gene4	7.1
Samp3	Root	Gene4	6.8
Samp4	Root	Gene4	6.9
Samp5	Root	Gene4	6.8
Samp6	Root	Gene4	6.9
Samp7	Leaf	Gene4	11.1
Samp8	Leaf	Gene4	10.8
Samp9	Leaf	Gene4	11.1
Samp10	Leaf	Gene4	10.8
Samp11	Leaf	Gene4	10.5
Samp12	Leaf	Gene4	11.1

Subsequently, utilizing the histogram plot tool with appropriate configurations provides expression distribution profiles for selected genes across different groups (Figure S5).

The tool also includes a function termed “explode matrix,” which expands matrix size by splitting elements within one column and duplicating values from another column in the same row (Figure 2C). This feature, referred to as “exploding,” amplifies matrix dimensions, as demonstrated in previous applications involving the transformation of gene ontology enrichment tables into network formats to visualize pathway‐gene relationships [33].

### Refined tool usage workflows

The operational procedures for each tool have been refined to enhance user interaction. Initially, users are prompted to define input parameters, such as specifying whether the input matrix is in the long or wide format if necessary and selecting between directly pasting data into a text area or using previously uploaded files. Following input submission, users engage the “Check Data” feature to validate adherence to predefined rules. For single matrices, validation includes checks for matrix legality (uniform row and column dimensions), absence of special characters in header rows (typically reserved for column names), absence of duplicate entries in the first column (typically reserved for row names in wide format), and numeric consistency for wide format matrices. Clear explanations are provided for detected errors, including error type, specific items causing issues, and their respective positions within the matrix. Users are empowered to rectify input data before proceeding with subsequent operations.

In cases where multiple matrices are involved, intermatrix relationships are scrutinized. For instance, in the context of a heatmap analysis comprising three matrices—heatmap data, row annotations, and column annotations—the system verifies that all items in the first column of annotation matrices align with corresponding entries in the heatmap data matrix. Continuous refinement of file validation logic is implemented based on user feedback to mitigate runtime failures effectively. Upon successful data validation, users can adjust additional parameters as desired to advance further on the tool page.

Parameters are logically grouped to simplify user interaction and presented in an accordion format. Groups lacking essential parameters remain folded, ensuring streamlined navigation. For tools like “heatmap,” where no essential parameters exist, all groups remain folded after data validation, allowing users to proceed with the submission promptly. Conversely, tools like “boxplot” feature essential parameters denoted by a red star, such as “X‐axis variable” and “Y‐axis variable,” which are initially expanded for convenient user selection. Additional parameters lacking star designation are considered optional, enabling users to focus solely on essential configurations initially. Post the run of the initial analysis, users can freely explore parameter explanations to experiment with adjustments and observe their effects.

Further enhancements include parameter optimization for clarity and functionality. Ambiguous parameters, such as those specifying data types, have been eliminated to reduce user confusion. Automated data type checks have been integrated into backend operations to enhance reliability. Parameters involving geometry ordering, such as “X‐axis variable order” now include data filtering capabilities. Users can select desired dropdown values to screen data or determine the plot layout, optimizing visualization output. Expanded parameter options encompass data preprocessing, statistical labeling, color customization, facet plots, and support for diverse output formats like interactive plots and PowerPoint presentations. Additionally, flexibility in column order and header names has been extended, broadening ImageGP's applicability beyond biological data sets to include data from any fields such as chemistry and physics—all predicated on structured matrix inputs.

### Expanded toolset

**Figure 3:**
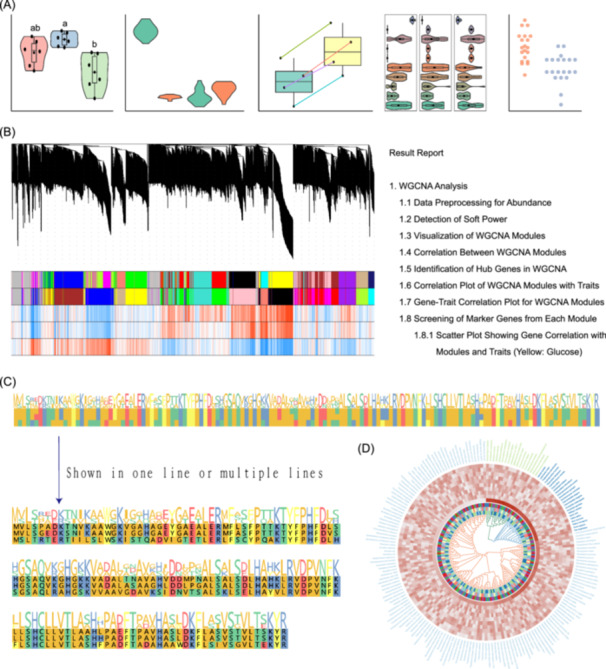
Representative visualization and analysis results. (A) Various configurations of box plots generated through parameter combinations, including single‐group, multiple‐group, paired‐line, facet plots, and beeswarm plots. (B) Weighted gene co‐expression network analysis results and corresponding comprehensive reports structured in eight sections. (C) Interactive plots for multiple sequence alignments, allowing dynamic layout adjustments. (D) Phylogenetic tree representation enriched with associated annotation heatmaps, facilitating the integration of qualitative and quantitative data for enhanced visualization.

The updated version of ImageGP features an enhanced array of tools with optimized parameter organization and increased flexibility. For instance, the box plot tool now supports various configurations such as single‐group, multiple‐group, pair‐lined, and facet box plots. Users can seamlessly transform these plots into violin plots, dot plots, jitter plots, or combinations thereof, and adjust the layout between vertical and horizontal orientations. Specific parameters are also provided for presenting single‐cell marker gene box plots (Figure 3A).

In the context of Linear discriminant analysis Effect Size analysis, users can directly input modified output to generate plots exclusively. Additionally, users could assign colors to each group and produce editable vector images with embedded text. Unlike the previous version, where all results are shown in one zipped file, users can now conveniently browse results through an online document enriched with text and images. This updated approach to result presentation not only facilitates comprehensive analysis with multiple steps and outcomes but also supports the integration of additional bioinformatics tools such as WGCNA and differential gene/protein expression analysis [34].

Beyond data transformation capabilities, this version introduces 10 new bioinformatics analysis tools, including WGCNA, limma differential expression analysis, multiple sequence alignment, reverse complement FATSA, RNA translation, motif search, FASTA extraction, points detection in specified areas, GXF to BED conversion, and CDS/protein sequence extraction from GXF files. Additionally, three interactive plot tools have been incorporated.

WGCNA analysis, for example, involves a structured process of eight steps designed for ease of use. Users simply paste or upload their expression data and initiate the analysis to receive comprehensive reports. Each report comprises eight sections corresponding to the analysis steps, featuring subsections with static or interactive visuals, tables, explanations, and options for downloading all results (Figure 3B). This reporting format can be extended to show results from cohesive workflows integrated by separate tools.

The multiple sequence alignment tool introduces interactive plots for the first time, allowing users to dynamically adjust layouts without recomputation. Interactive features enable users to hover over plot elements for detailed information (Figure 3C). Notably, the Circle phylogenetic tree tool supports phylogenetic analysis based on Newick format input, incorporating various annotations (Figure 3D). Phylogenetic trees are pivotal for organizing biological diversity knowledge, structuring classifications, and providing evolutionary insights. Users can enrich these trees with attribute matrices to set branch and node colors, integrating qualitative and quantitative information for enhanced data visualization.

### Personalized user environment

While ImageGP does not mandate login for usage, registered users could gain access to personalized features to manage large data sets more efficiently. For example, users may encounter browser crashes when directly pasting large input matrices into the web page text area, potentially leading to suboptimal user experiences. To mitigate this issue, a personalized user space has been implemented for registered users, consisting of two main components: File Management and Tools Records. Registration is straightforward and free of charge.

In the File Management section, users can upload, copy, move, rename files, and organize directories. Uploaded files can be selected for use in tool pages, with a preview feature displaying the initial five lines of text content. For files exceeding 500 characters per line, only the first 500 alphabetic characters in each line are shown. The selection of directories displays the first five files/folders contained within, allowing users to verify file choices efficiently without overwhelming the web page with excessive content. However, the complete file content is utilized during subsequent parameter selection and actual analysis phases.

Within the Tools Records section, logged‐in users can review submission times, execution statuses, and results of each submitted job. Collaboration is facilitated through the ability to share results with collaborators. Users can adjust parameters based on previous selections, conduct reanalysis of jobs either retaining previous parameters or specifying new ones, and save results into distinct folders as either updates to existing jobs or entirely new analyses. This functionality enhances user control and workflow management across multiple analysis sessions.

### Improved user support with streamlined error reporting

While we conduct preliminary checks on input data before submission, unforeseen errors may still occur due to user‐defined parameters or specific data content like empty values or symbol conflicting. These instances are categorized as operational challenges for online tools. To address these issues proactively, based on accumulated experience, we implement rigorous data and parameter validation processes. However, users may still meet runtime errors. Those users unfamiliar with programming may struggle to provide comprehensive information for debugging purposes.

To streamline error resolution, a “Request for Help” button is now integrated into the result page. This feature automatically detects errors during program execution and prompts users to submit error logs directly to our team. Upon receiving these logs, our developers promptly initiate debugging protocols to refine the program code and address identified issues. Users who opt to leave their contact emails could receive responses within 1–3 days, containing pertinent debug information and resolutions.

This enhancement not only simplifies the error reporting process for users but also enables continuous program optimization based on real‐world feedback. As a result, ImageGP 2 remains agile in addressing evolving data complexities and user requirements through iterative updates and enhancements.

### The R package ImageGP

ImageGP 2 represents a reimagined web server that integrates the new R package ImageGP pivotal to the functionality of various analysis and visualization tools within the platform.

Previously, the online version of ImageGP utilized bash scripts to dynamically generate R scripts based on user inputs, resulting in repetitive code logic across scripts. This approach complicated code revision for debugging and the inclusion of new functionalities. Moreover, users unfamiliar with bash scripting faced challenges in executing these scripts. In the redesigned version, we have transitioned to using pure R code to handle user inputs and parameter validation. Similar functional blocks of code have been modularized into R functions, totaling 96 functions including 12 primary plotting functions. These functions encompass data transformation, logical checks, and attribute mapping, systematically employed across all visualization tools and other operational contexts. Consequently, bug fixes applied to individual functions propagate throughout all tools, enhancing efficiency and maintenance.

The R package ImageGP retains the same parameters as its online counterpart. Each data visualization tool generates an R script tailored to user inputs and parameters. Users have the option to download these scripts, open them in RStudio or other R Integrated Development Environments, and customize file paths and output prefixes for local execution. This capability offers several advantages: first, it enables users to simulate data copies with identical headers, select parameters online, generate visualizations, and subsequently replace file locations locally to produce real results, ensuring data privacy. Second, users can introduce additional tuning parameters directly into the local R script for customized analyses. Third, the local script facilitates batch plot generation through iterative processes.

This enhanced integration of the R package ImageGP not only enhances user flexibility and data privacy but also empowers advanced users to extend functionality and optimize analyses beyond standard parameters available online.

## DISCUSSION

ImageGP was originally developed to enhance data visualization capabilities for researchers, which has proven beneficial. However, it is crucial for users to recognize that visualizations containing numerous data points can lead to misinterpretations. Poorly designed visualizations may introduce biases or confusion. A simple graph might fail to capture attention or convey significant insights, whereas an elaborate visualization could obscure the intended message or offer profound clarity.

In recent years, there has been an increasing demand for effective visual representation of information, especially in scientific research contexts. Successful data visualization transcends mere graphical depiction by requiring clear objectives that drive design choices. Researchers must determine what specific aspects of the data they wish to visualize. This involves decisions on geometric elements (e.g., points, lines, bars), mapping data columns to aesthetic attributes such as color, shape, and size, applying statistical transformations, and specifying the coordinate system for the plot. Techniques like faceting enable the visualization of different data subsets. The integration of these components defines the graphical output.

When designing ImageGP, our approach guides users through selecting the plot type and configuring data attributes like x‐axis, y‐axis, color, size, and shape. This approach aims to familiarize users with the data visualization process and facilitate the interpretation of visual results. ImageGP also provides users with the flexibility to experiment with various visualization types to identify the most suitable one for their needs.

ImageGP2 represents a substantial evolution from its predecessor. Our ongoing updates focus on expanding functionality and enhancing user accessibility. Currently, we offer text and video tutorials along with training courses to reduce usability barriers. Looking ahead, our development efforts for ImageGP will concentrate on two primary objectives: first, transforming ImageGP into a computational platform that simplifies the transition from command‐line tools to online tools, thereby broadening accessibility for researchers. Second, integrating individual tools into workflows that enable users to initiate analyses directly from raw data, such as raw sequence data in FASTQ format, progressing seamlessly through mapping, quantification, and subsequent visualizations.

## CONCLUSION

In summary, ImageGP 2 emerges as a versatile tool at the forefront of biomedical research, facilitating seamless integration between data matrices and visual representations. Its enhanced capabilities promise to empower researchers in leveraging big biological data for transformative discoveries and applications in various scientific domains.

## METHODS

ImageGP 2 is hosted in a high‐performance computing server with 180 threads, 386 GB memory, and 15 TB storage to deal with 2000 jobs each day. ImageGP 2 is implemented as a web application using JavaScript and HTML for front‐end development. The core JavaScript libraries used include Vue.js (https://vuejs.org) for the main frame, vis.js (https://visjs.org) for network display, echarts (https://echarts.apache.org), plotly.js (https://plotly.com/), and D3.js (https://d3js.org/) for interactive charts (like multiple sequence alignment, phylogenetic tree visualization, maps). The backend data transporting was conducted using the high‐level web framework Django (https://www.djangoproject.com). The MySQL open‐source data management system is utilized for saving and accessing table data. All submitted jobs are managed by the distributing system Celery (https://docs.celeryq.dev/en/stable/index.html) scheduled in two queues (data analysis queue and data visualization queue) with Redis as the backend. Most data visualization is generated based on the R package ImageGP which mainly depends on the ggplot2, WGCNA, and limma packages [32, 34, 35]. The R packages plotly and eoffice are used to transfer picture objects to interactive plots or Microsoft PowerPoint formats. Most data format transforming and sequence processing are dealt with using Python scripts.

## AUTHOR CONTRIBUTIONS

Tong Chen wrote the manuscript with all figures. Mei Yang drafted the first two figures. Tao Chen, Mei Yang, Siqing Fan, Minglei Shi, Buqing Wei, Huijiao Lv, Wandi Cao, Chongming Wang, Jianzhou Cui, Jiwen Zhao, Yilai Han, Jiao Xi, and Ziqiang Zheng tested the tools and gave useful feedback. Tong Chen, Yong‐Xin Liu, and Luqi Huang supervised and funded this project, and revised the manuscript. All authors have read the final manuscript and approved it for publication.

## CONFLICT OF INTEREST STATEMENT

Tong Chen and Yong‐Xin Liu hold the position of Editor‐in‐Chief for *iMeta* and are blinded from peer review and decision‐making for the manuscript.

## ETHICS STATEMENT

No animals or humans were involved in this study.

## Supporting information


**Figure S1:** Density plot showing the expression distribution profile for Gene1 in all samples.
**Figure S2:** Combine these two matrices to create a merged data set. All configured parameters are highlighted in yellow.
**Figure S3:** Visualizing the expression distribution profile of each gene across different conditions.
**Figure S4:** Transfer wide matrix to long matrix.
**Figure S5:** Displaying expression distribution profiles for selected genes across different groups.

## Data Availability

This paper does not generate any new data. ImageGP 2 could be accessed at https://www.bic.ac.cn/BIC/#/. The R package ImageGP is saved in https://github.com/Tong-Chen/ImageGP and https://gitee.com/ct5869/ImageGP. Supplementary materials (figures, tables, scripts, graphical abstract, slides, videos, Chinese translated version, and update materials) may be found in the online DOI or iMeta Science http://www.imeta.science/.
