# Supplementary material for: ImageGP 2 for enhanced data visualization and reproducible analysis in biomedical research
Source: Imeta. 2024 Sep 12;3(5):e239. doi: 10.1002/imt2.239 (PMC11487545; doi:10.1002/imt2.239)
Supplement: Supplementary file 1 — Figure S1: Density plot showing the expression distribution profile for Gene1 in all samples. Figure S2: Combine these two matrices to create a merged data set. All configured parameters are highlighted in yellow. Figure S3: Visualizing the expression distribution profile of each gene across different conditions. Figure S4: Transfer wide matrix to long matrix. Figure S5: Displaying expression distribution profiles for selected genes across different groups. [file IMT2-3-e239-s001.docx]

**Supporting information to:**

**ImageGP 2 for Enhanced Data Visualization and Analysis in Biomedical Research**

Running title: Enhanced Biomedical Data Visualization with ImageGP 2

Tong Chen^1^*, Yong-Xin Liu^2^*, Tao Chen^3^, Mei Yang^1^, Siqing Fan^4^, Minglei Shi^5^, Buqing Wei^6^, Huijiao Lv^7^, Wandi Cao^8^, Chongming Wang^9^, Jianzhou Cui^10,11,12^, Jiwen Zhao^13^, Yilai Han^14^, Jiao Xi^15^, Luqi Huang^1^*

^1^State Key Laboratory for Quality Ensurance and Sustainable Use of Dao-di Herbs, National Resource Center for Chinese Materia Medica, China Academy of Chinese Medical Sciences, Beijing 100000, China

^2^Genome Analysis Laboratory of the Ministry of Agriculture and Rural Affairs, Agricultural Genomics Institute at Shenzhen, Chinese Academy of Agricultural Sciences, Shenzhen 518120, China

^3^Northwest Institute of Plateau Biology, Chinese Academy of Sciences, Qinghai, China

^4^Lushan Botanical Garden, Chinese Academy of Sciences, Jiujiang 332900, China

^5^School of Life Course and Population Sciences, King's College London, UK

^6^State Key Laboratory of Efficient Utilization of Arid and Semi-arid Arable Land in Northern China, the Institute of Agricultural Resources and Regional Planning, Chinese Academy of Agricultural Sciences, Beijing 100081, China

^7^Key Laboratory of Human Disease Comparative Medicine, National Health Commission of China (NHC), Institute of Laboratory Animal Science, Chinese Academy of Medical Sciences, Peking Union Medicine College, Beijing, China

^8^Nanjing Agricultural University, Nanjing, China

^9^College of Resources and Environment, Huazhong Agricultural University, Wuhan 430070, Hubei, China

^10^Immunology Translational Research Program, Yong Loo Lin School of Medicine, National University of Singapore, Singapore, Singapore

^11^Immunology Program, Life Sciences Institute, National University of Singapore, Singapore, Singapore

^12^NUS-Cambridge Immunophenotyping centre, National University of Singapore, Singapore, Singapore

^13^State Key Laboratory of Crop Stress Resistance and High-Efficiency Production, College of Agronomy, Northwest A&F University, Yangling, Shaanxi 712100, P. R. China

^14^Department of Neurology, Xuanwu Hospital Capital Medical University, National Center for Neurological Disorders, Beijing, China

^15^College of Natural Resources and Environment, Northwest A&F University, Yangling, Shaanxi, China

*Correspondence: chent@nrc.ac.cn/chentong_biology@163.com (Tong Chen), huangluqi01@126.com (Luqi Huang), and [liuyongxin@caas.cn](mailto:liuyongxin@caas.cn) (Yong‐Xin Liu)

**
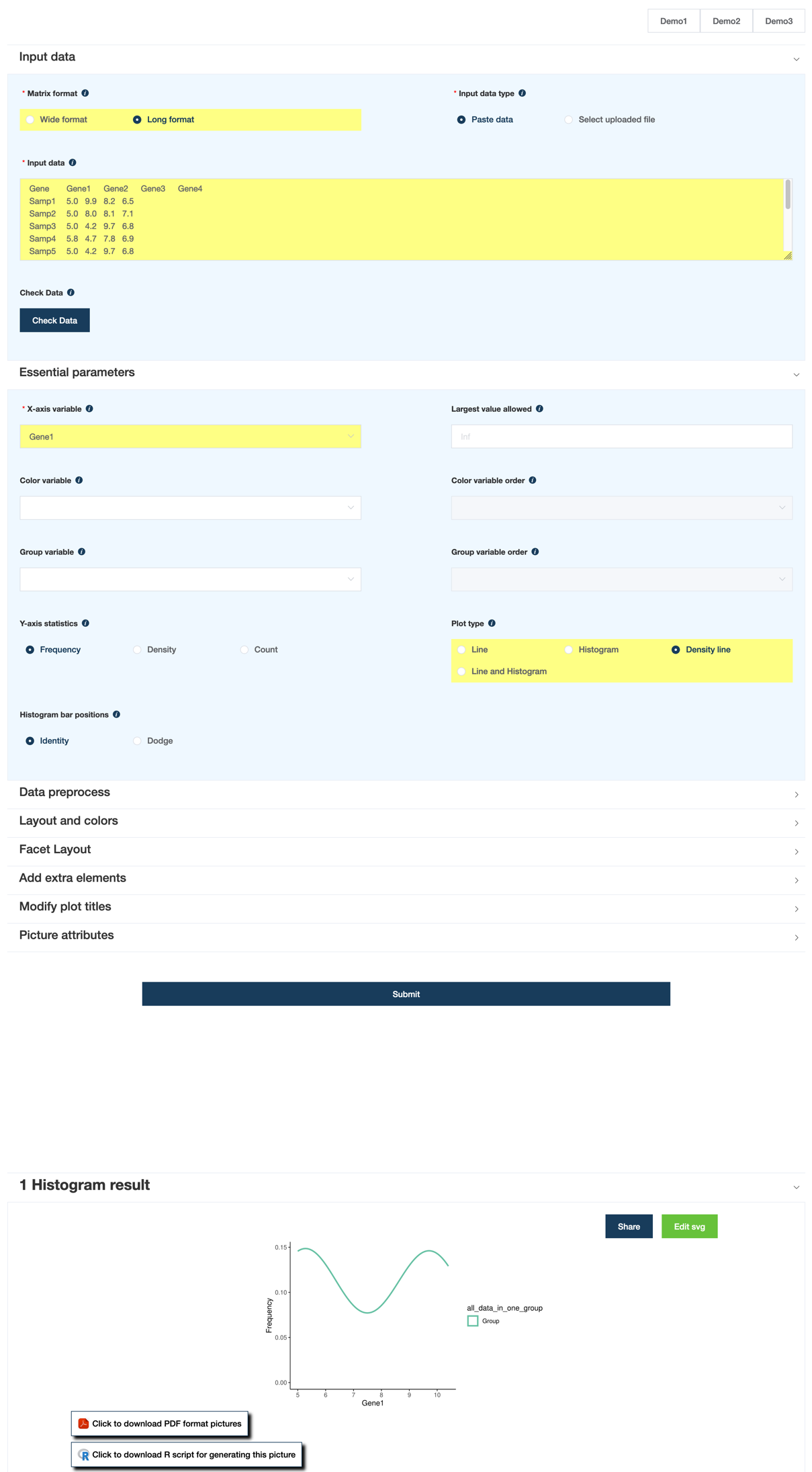
**

**Figure S1** **Density plot showing the expression distribution profile for Gene1 in all samples.** All configured parameters are highlighted in yellow.


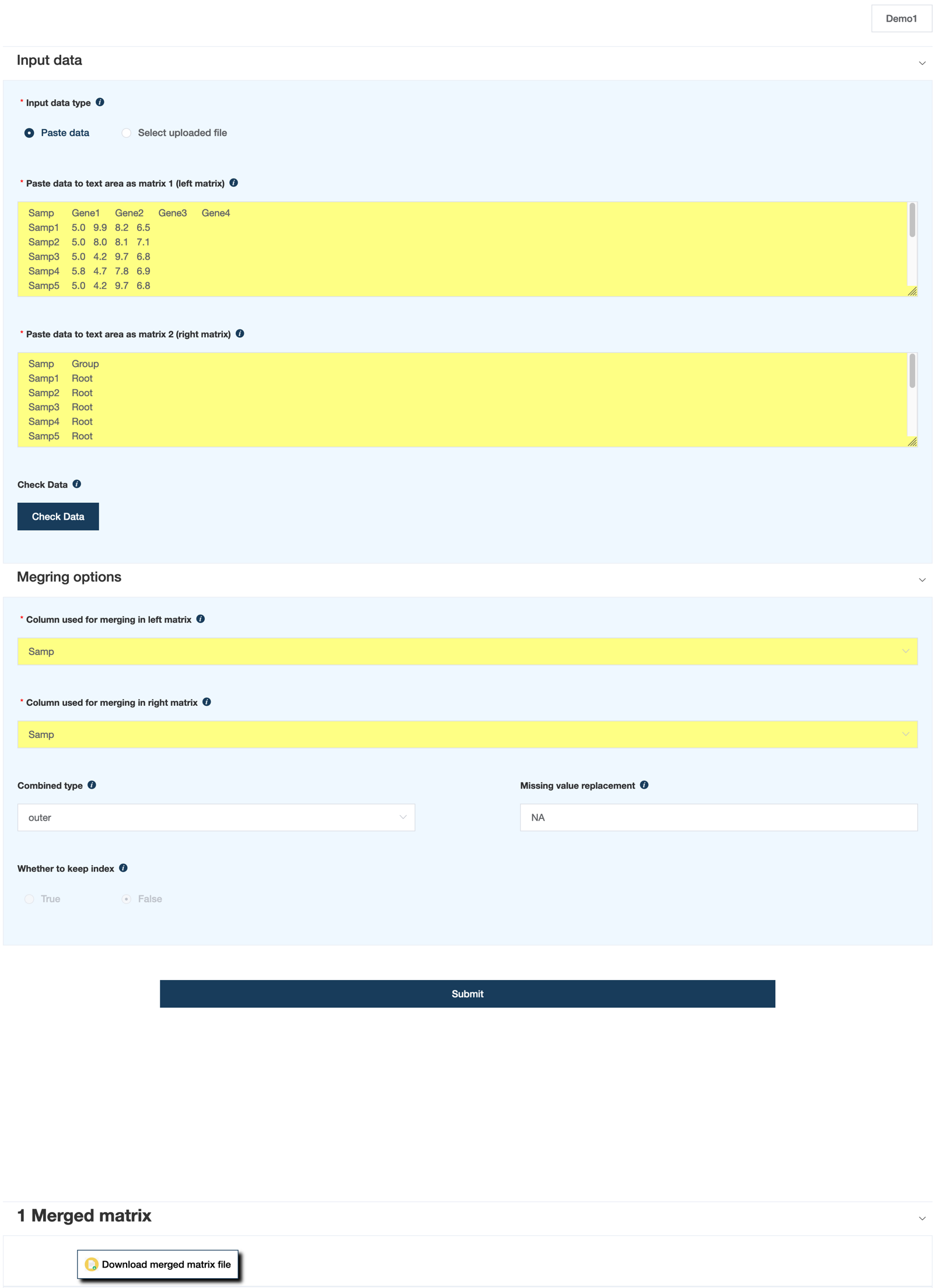


**Figure S2** **Combine these two matrices to create a merged dataset.** All configured parameters are highlighted in yellow.


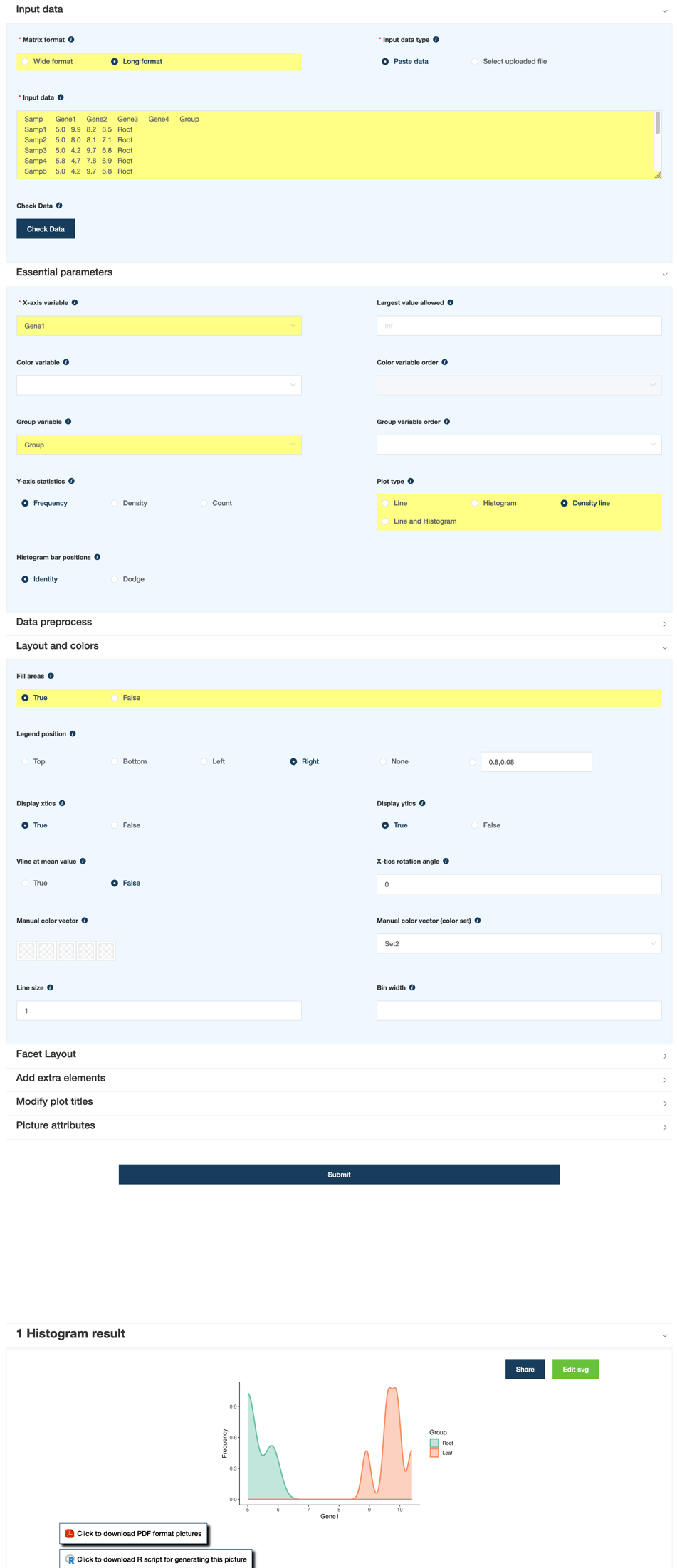


**Figure S3** **Visualizing the expression distribution profile of each gene across different conditions.** All configured parameters are highlighted in yellow.


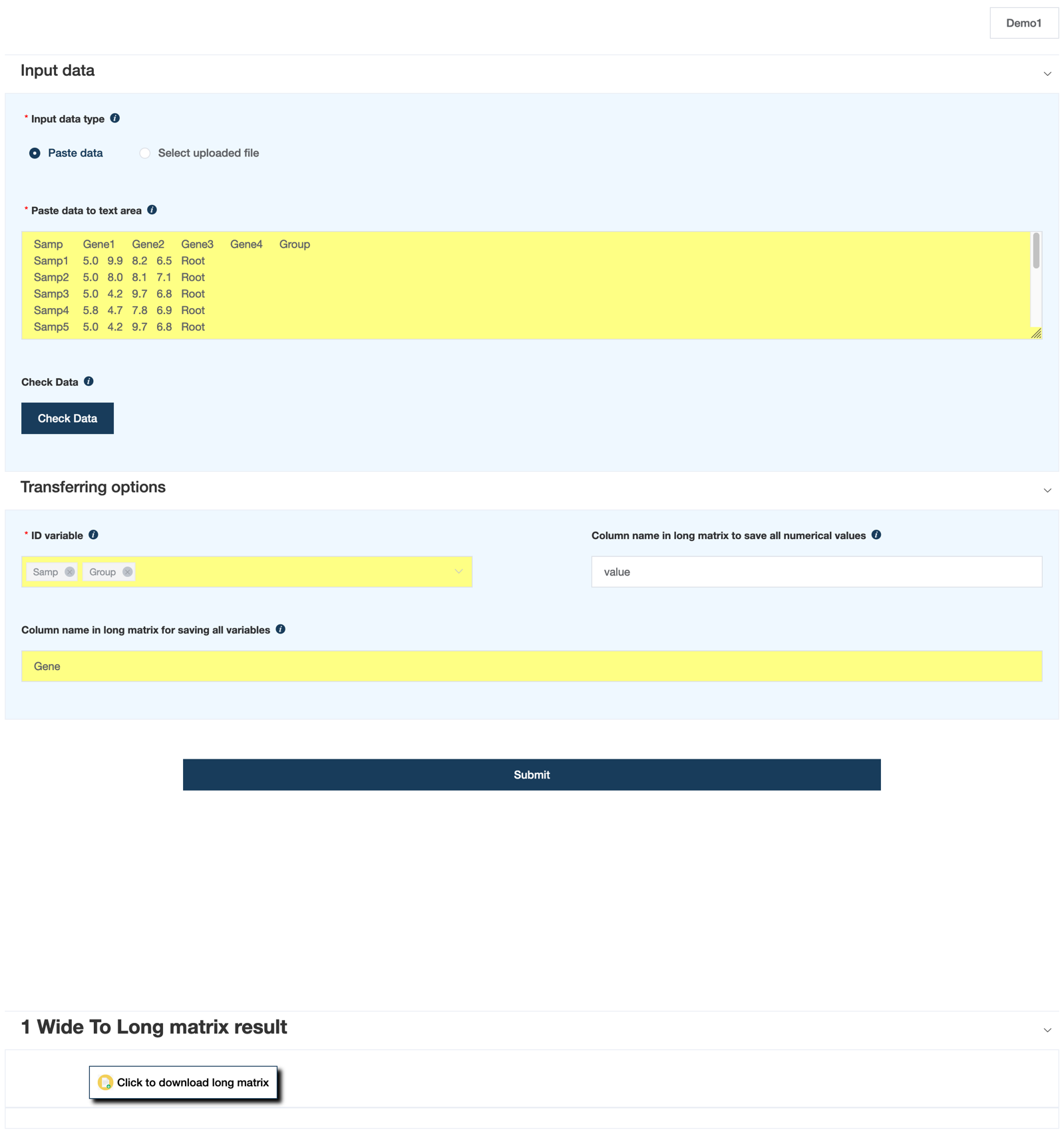


**Figure S4** **Transfer wide matrix to long matrix.** All configured parameters are highlighted in yellow.


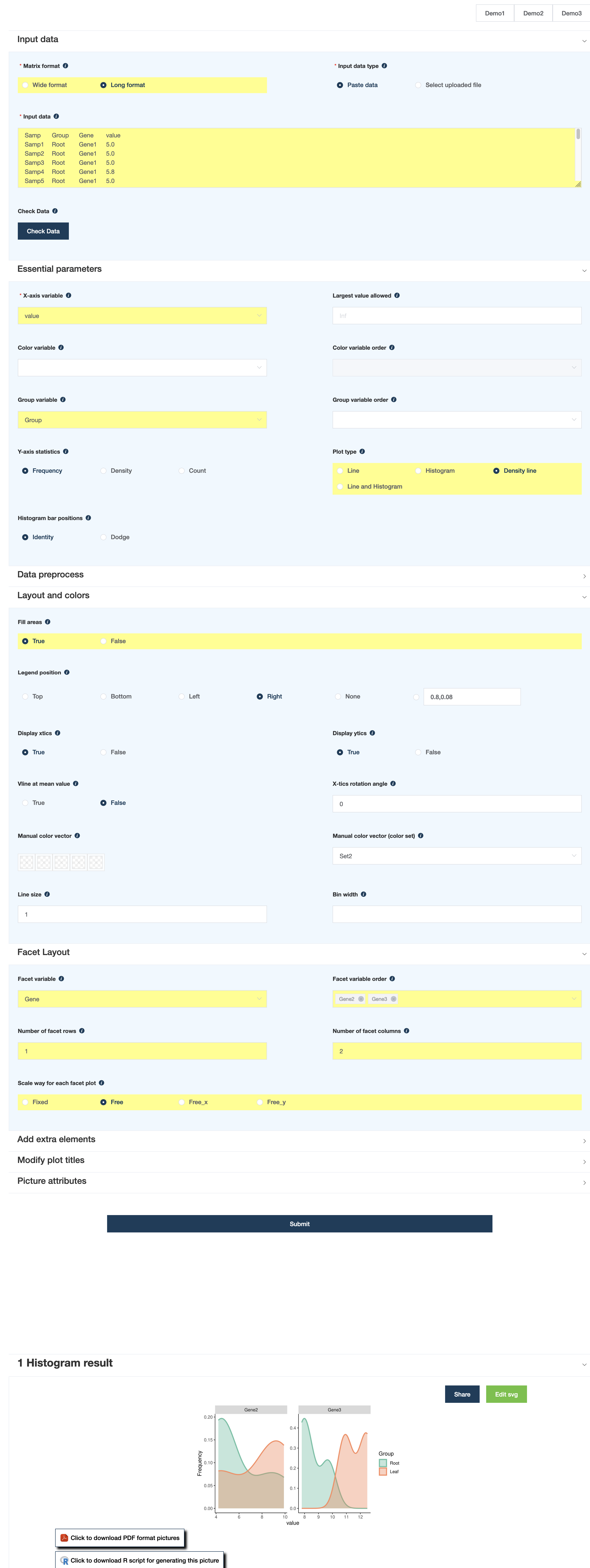


**Figure S5** **Displaying expression distribution profiles for selected genes across different groups.** All configured parameters are highlighted in yellow.

(B)
